# Integrated analysis of single-cell RNA-seq, bulk RNA-seq, Mendelian randomization, and eQTL reveals T cell-related nomogram model and subtype classification in rheumatoid arthritis

**DOI:** 10.3389/fimmu.2024.1399856

**Published:** 2024-06-19

**Authors:** Qiang Ding, Qingyuan Xu, Yini Hong, Honghai Zhou, Xinyu He, Chicheng Niu, Zhao Tian, Hao Li, Ping Zeng, Jinfu Liu

**Affiliations:** ^1^ The First School of Clinical Medicine, Guangxi Traditional Chinesen Medical University, Nanning, China; ^2^ Gynecology Department, The First People’s Hospital of Guangzhou, Guangzhou, China; ^3^ Faculty of Orthopedics and Traumatology, Guangxi University of Chinese Medicine, Nanning, China; ^4^ Department of Orthopedics and Traumatology, The First Affiliated Hospital of Guangxi University of Traditional Chinese Medicine, Guangxi, China

**Keywords:** rheumatoid arthritis, single-cell RNA sequencing, Mendelian randomization, bulk RNA sequencing, combined biomarkers, machine learning, T cells

## Abstract

**Objective:**

Rheumatoid arthritis (RA) is a systemic disease that attacks the joints and causes a heavy economic burden on humans worldwide. T cells regulate RA progression and are considered crucial targets for therapy. Therefore, we aimed to integrate multiple datasets to explore the mechanisms of RA. Moreover, we established a T cell-related diagnostic model to provide a new method for RA immunotherapy.

**Methods:**

scRNA-seq and bulk-seq datasets for RA were obtained from the Gene Expression Omnibus (GEO) database. Various methods were used to analyze and characterize the T cell heterogeneity of RA. Using Mendelian randomization (MR) and expression quantitative trait loci (eQTL), we screened for potential pathogenic T cell marker genes in RA. Subsequently, we selected an optimal machine learning approach by comparing the nine types of machine learning in predicting RA to identify T cell-related diagnostic features to construct a nomogram model. Patients with RA were divided into different T cell-related clusters using the consensus clustering method. Finally, we performed immune cell infiltration and clinical correlation analyses of T cell-related diagnostic features.

**Results:**

By analyzing the scRNA-seq dataset, we obtained 10,211 cells that were annotated into 7 different subtypes based on specific marker genes. By integrating the eQTL from blood and RA GWAS, combined with XGB machine learning, we identified a total of 8 T cell-related diagnostic features (MIER1, PPP1CB, ICOS, GADD45A, CD3D, SLFN5, PIP4K2A, and IL6ST). Consensus clustering analysis showed that RA could be classified into two different T-cell patterns (Cluster 1 and Cluster 2), with Cluster 2 having a higher T-cell score than Cluster 1. The two clusters involved different pathways and had different immune cell infiltration states. There was no difference in age or sex between the two different T cell patterns. In addition, ICOS and IL6ST were negatively correlated with age in RA patients.

**Conclusion:**

Our findings elucidate the heterogeneity of T cells in RA and the communication role of these cells in an RA immune microenvironment. The construction of T cell-related diagnostic models provides a resource for guiding RA immunotherapeutic strategies.

## 1 Introduction

Rheumatoid arthritis (RA) is a commonly occurring autoimmune disease that affects 0.5–1% of the world’s population (1). The mechanism of RA has not yet been elucidated, and an effective cure is still lacking. It’s reported that chronic and persistent synovial inflammation is a typical pathological feature of RA. Many abnormal immune cells continue to invade the affected joints of RA patients, forming an extremely complex regulatory network. Abnormal regulation promotes pannus formation and injury to the bone and cartilage (2). T cells can be recruited through blood and lymphatic circulation into the synovial membrane of a joint and interact with dendritic cells, macrophages and B cells, which is one of the most important factors in triggering RA (3, 4). CD4+ T cells are the primary inflammatory cells that invade synovial tissue and participate in the pathogenesis of RA (5). Ectopic germinal centers in the synovium, which are considered to be a characteristic of RA, require CD8+ T cells for their development (6). In addition, CD8+ T cell activation is promoted by antigens presented by other cells, which can exacerbate inflammation in RA (7). However, due to methodological limitations, the potential mechanisms by which T cells influence RA and their potential applications for RA diagnosis and treatment have not been extensively studied.

Expression quantitative trait loci (eQTL) is a region of chromosomes that explains how genetic variation is correlated with expression levels of specific genes (8). It’s reported that genetic factors are crucial in RA. Genome-wide association studies (GWAS) have successfully identified several sites that are susceptible to RA (9). With the advent of Mendelian randomization, researchers have been able to more scientifically identify disease risk genes by integrating eQTL and GWAS, which can help reveal biological pathways from genetic determinants to transcriptome signatures and phenotypic outcomes.

**Figure 1:**
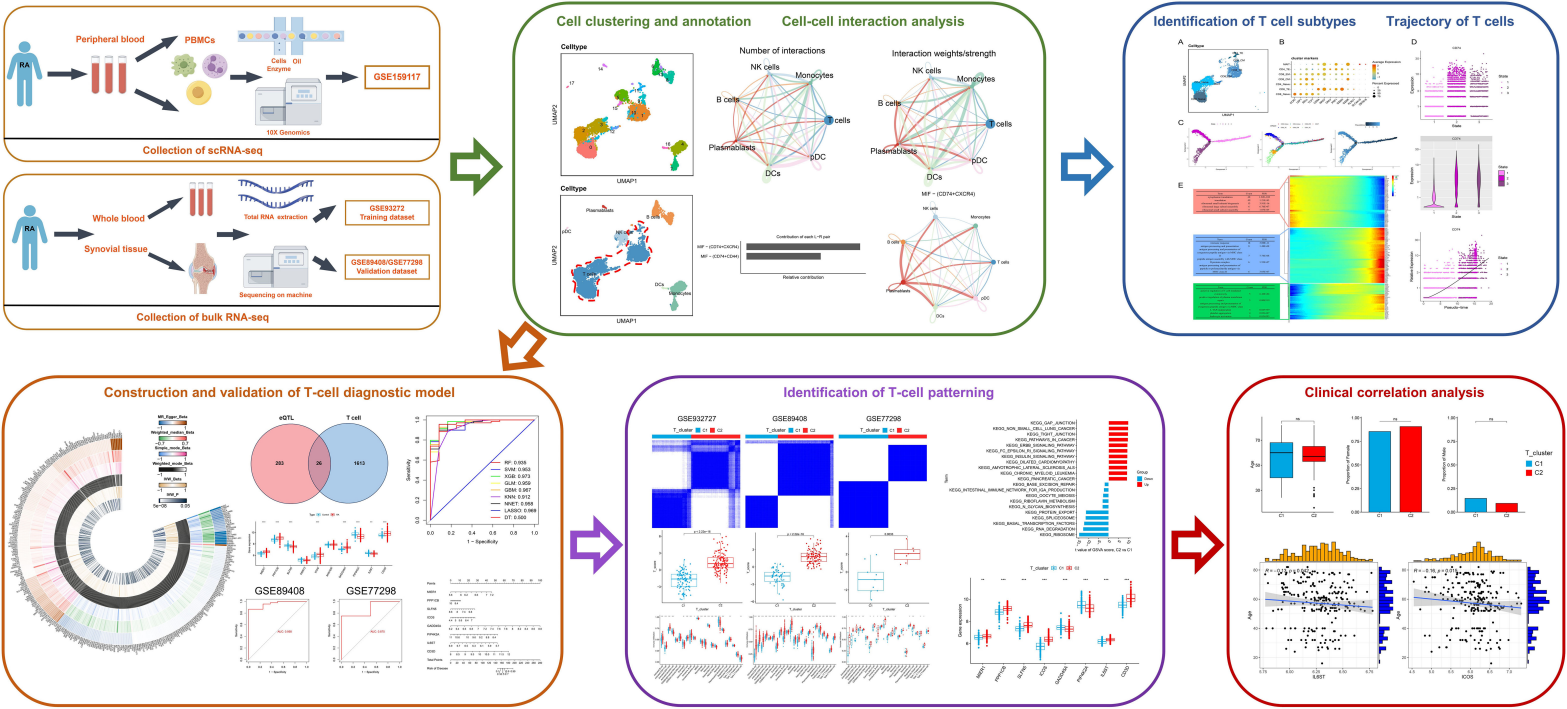
The workflow of this study.

Single-cell RNA sequencing (scRNA-seq), which can explore gene expression profiles at single-cell resolution, is becoming a powerful tool used in human disease research. This method can reveal the heterogeneity of cells, the development process, and the relationship between cells, which is helpful to further understand the pathogenesis of RA (10). Although bulk RNA-seq has limitations with capturing cell-cell interactions, they complement results obtained from the scRNA-seq analysis, further clarifying gene expression profiles associated with RA. Therefore, we aimed to identify T cell-related diagnostic features and clusters in RA by integrating multiple datasets (eQTL, GWAS, scRNA-seq, and bulk RNA-seq) combined machine learning. The resulting T cell-related diagnostic features were used to construct a valuable RA diagnostic model that supports accurate diagnoses and personalized treatment strategies for RA (**Figure 1**).

## 2 Materials and methods

### 2.1 Data preparation

EQTL data were collected from the IEU OpenGWAS project (https://gwas.mrcieu.ac.uk/) which contained 19942 genes (**Supplementary File 1**). RA GWAS data from FinnGen R10 (https://www.finngen.fi/fi) included 13,261 RA patients and 262,844 healthy individuals. All data were from European-ancestry individuals.

scRNA-seq and microarray datasets were obtained from the Gene Expression Omnibus (GEO) database. The scRNA-seq dataset GSE159117 included data from peripheral blood mononuclear cells (PBMCs) from a patient with RA. The microarray datasets included dataset files GSE93272 (platform: GPL570), GSE89408 (platform: GPL11154) and GSE77298 (platform GPL570), which included data for whole blood from 232 RA patients and 43 healthy individuals, synovial tissues from 152 RA patients and 28 healthy individuals and synovial tissues from 16 RA patients and 7 healthy individuals respectively. GSE93272 was used as the training dataset, GSE89408 and GSE77298 were used as validation datasets.

### 2.2 Mendelian randomization analysis

In this study, eQTL data were used as the exposure and RA GWAS data as the outcome to identify genes associated with RA in R software (version 4.3.2). We selected SNPs significantly correlated with RA (*P <*5×10^-8^). The clumping process (involving an r^2^<0.01 and a clumping distance of 10,000 kb) was conducted to assess the linkage disequilibrium (LD) between the included SNPs (11). Beta coefficients per allele, p-values and standard errors for the each identified SNP were extracted from the RA GWAS dataset. Instrumental variables (IVs) significantly associated with the outcome phenotype were removed (*P <*5×10^-8^). We then harmonized the SNP instruments by calibrating the directions of the alleles for both Exposure-SNPs and Outcome-SNPs and removing SNPs with ambiguous palindromic sequences. An F statistic (F = beta^2^_exposure_/SE^2^_exposure_) greater than ten is used as a threshold to reduce bias caused by weak IVs (12). The “TwoSampleMR” package (version 0.5.10) and Inverse-variance weighted (IVW) method were used for two-sample MR analysis. Horizontal pleiotropy was examined by whether the MR-Egger intercept deviated significantly from 0. Cochran’s Q statistic and corresponding p-values were calculated to determine heterogeneity, a *P >*0.05 indicated no significant heterogeneity. Finally, the reliability of the results and potential impact of SNPs were evaluated via Leave-one-SNP-out analysis.

### 2.3 scRNA-seq data processing and identification of cell types

This study analyzed PBMCs from one patient with RA. The Seurat package’s CreateSeuratObject function was used to convert PBMCs into a Seurat object in R software, and then quality control was performed on each cell according to gene number 200–6000, UMI count >1000, and mitochondrial gene percentage <10%. Subsequently, the data was normalized and 2000 highly variable genes were screened for further analysis. Principal component analysis (PCA) was performed to reduce the dimensions, and the top 30 principal components were selected for further analysis with the resolution set to 1.0. PBMC clustering was conducted using “FindNeighbors” and “FindClusters”. Furthermore, data visualization was using uniform manifold approximation and projection (UMAP). Based on a threshold of *P* < 0.05, log_2_FC > 0.25, “FindAllMarkers” was used to identify differentially expressed genes in each cluster. Based on the unique marker genes in the study, we analyzed the expression of these marker genes in different clusters and annotated the cells (10). T cells were extracted for downstream analysis.

### 2.4 Cell-cell communication analysis

The CellChat package was used to explore the communication mode between different immune cells in the immune microenvironment of RA (13). Specific ligand and receptor interactions and important signaling pathways between T cells and other immune cells were also identified.

### 2.5 Analysis of pseudo-time trajectories gene ontology enrichment

To further study how T cells affect the RA microenvironment, we analyzed T cell developmental trajectories using the monocle package (14). The function “reduceDimension” was used to reduce dimensions. We also used the “plot_cell_trajectory”, “plot_pseudotime_heatmap “ and “plot_genes_branched_pseudotime” functions for identifying cell differentiation trajectories, visualizing differential genes and displaying gene changes over time. Subsequently, we performed Gene ontology (GO) enrichment analysis for the differential genes of T cell populations in different differentiation states by DAVID, and retained the results belonging to biological processes (BP).

### 2.6 Identification of T cell-related diagnostic features

We intersected RA-related genes obtained by MR with T cell marker genes. We randomly used 70% of the samples of GSE93272 dataset as the training group to build the classification model, and the remaining 30% of the samples were used as the validation group to validate the model. Various algorithms were used for model construction, including the Least absolute shrinkage and selection operator (LASSO), Extreme gradient boosting (XGB), Gradient boosting machine (GBM), Generalized linear model (GLM), Neural network (NNET), Support vector machine (SVM), K-nearest neighbors (KNN), Random forest (RF) and Decision tree (DT) were implemented in R (version 3.6.1) using the “caret”, “DALEX”, “ggplot2”, “randomForest”, “kernlab” and “xgboost” packages in the training group. The models were used to analyze the importance of intersection genes and output the top 10 key diagnostic genes based on the importance score obtained from each algorithm. The receiver operating characteristic (ROC) curve was generated using the “pROC” package. Based on the residuals box plot, using the residuals reverse cumulative distribution and ROC curve, we selected the optimal model validated by the ROC of validation datasets (GSE89408 and GSE77298) and T cell-related features. Subsequently, Wilcoxon tests were used to screen for T cell-related diagnostic features with *P* < 0.05 between RA and healthy individuals in GSE93272.

### 2.7 Construction and verification of a nomogram model

Based on T cell-related diagnostic features, the “rms” package was used to construct a nomogram model for predicting RA risk. “Calibration curve”, “decision curve analysis (DCA)” and “clinical impact curve” were used to evaluate and verify the accuracy and efficiency of this model. The external datasets, GSE89408 and GSE77298, were used for validation.

### 2.8 Consensus clustering analysis and PCA

Considering the differences in the expression of T cell-related diagnostic features, we used the “ConsensusClusterPlus” package to conduct consensus cluster analysis (15) on RA patients in the GSE93272 dataset to identify distinct T cell patterns. Additionally, RA patients from the GSE89408 and GSE77298 datasets served as validation for the clustering results. The consensus clustering parameters were set as follows: reps = 50, pItem = 0.8, pFeature = 1, clusterAlg = “km” and distance = “euclidean”. We evaluated the cumulative distribution function (CDF) curve for 9 subtypes to determine the optimal cluster number. To assess the reliability of the clustering, PCA scatter plots were generated using the clustering result data.

### 2.9 Gene set variation analysis (GSVA) analysis

GSVA is used to assess changes in the activity of pathways and functions in which gene sets are located. We conducted GSVA analysis of different T cell clusters in RA to identify differentially expressed pathways through GSVA package, where. The “c2.cp.kegg.symbols” file was obtained from the MSigDB P < 0.05 suggested that the pathways were significantly different.

### 2.10 Analysis of immune cell infiltration

Single-sample gene set enrichment analysis (ssGSEA) was performed using the GSEABase and GSVA software packages based on markers for 22 immune cells. This analysis involved ranking gene expression levels within the samples and summing these levels to quantify the abundance of immune cells in each sample, thereby generating a file of immune cell infiltration results. Subsequently, the clustering result and immune cell infiltration result files were analyzed using the “limma”, “reshape2” and “ggpub” packages to construct boxplots. Additionally, the correlation between the expression of T cell-related diagnostic features and immune cells was determined using correlation test. The T cell scores of different T cell clusters were calculated using the PCA algorithm to evaluate the relationship between the two clusters.

### 2.11 Clinical correlation analysis

To further explore the clinical correlation of the eight T cell-related diagnostic features, the age and sex characteristics of all samples in GSE93272 were extracted. Wilcoxon test was used to determine the sex and age distribution of T cell clusters in RA. Age-related correlations between T cell-related diagnostic features and RA were analyzed using Spearman.

### 2.12 Statistical analysis

All parametric analyses were performed using two-tailed tests. The Wilcoxon test was used to compare the differences between the two independent groups of samples, linear regression analyses were used to explore correlations between T cell-related diagnostic features and immune cells. Calculation and visualization of the area under the curve (AUC) using the pROC package. P < 0.05 is the threshold for significance.

## 3 Results

### 3.1 Identification of cell types in RA

**Figure 2:**
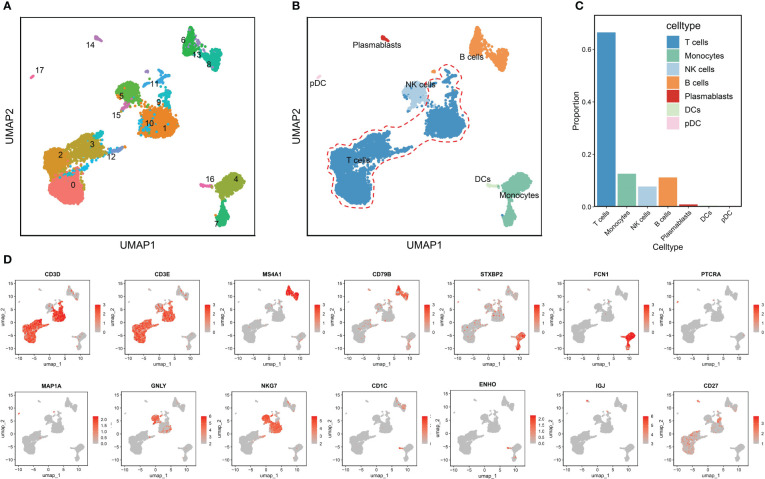
Annotation of clusters and subpopulations of cells in the RA scRNA-Seq data. **(A)** UMAP of 18 cell clusters. **(B)** 10,211 cells were labeled by cell type in the UMAP analysis. **(C)** The proportions of different cell types. **(D)** Marker genes for T cells, B cells, monocytes, pDCs, NK cells, DCs, and plasmablasts are presented in UMAP results.

The RA dataset contained 10,211 cells divided into 18 clusters (**Figure 2A**), which annotated as follows: T cells characterized by high expression of CD3D and CD3E (clusters 0,1,2,3,9,10,11 and12); monocytes with high expression of STXBP2 and FCN1 (cluster 4 and 7), NK cells with GNLY and NKG7 as marker genes (clusters 5and15); B cells marked by MS4A1 and CD79B (clusters 6,8and13); plasmablasts characterized by high expression of IGJ and CD27 (cluster 14); dendritic cells (DCs) (cluster 16) and pDCs (cluster 17) marked by CD1C, ENHO and PTPRA, MAP1A, respectively (**Figure 2B**). **Figure 2C** shows that T cells were significantly more abundant in the RA immune microenvironment compared to other immune cell types. Therefore, we therefore investigated the role of T cells in RA. **Figure 2D** displays the significant marker genes for each cell type in the PBMCs.

### 3.2 Analysis of cell-cell communication in the RA microenvironment

**Figure 3:**
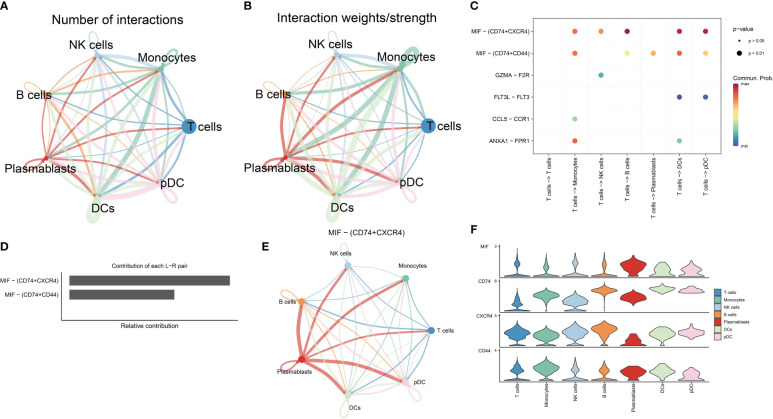
Analysis of cell-cell communication in the RA immune microenvironment. **(A, B)** The number and strength of interactions in cellular communication networks. **(C)** Bubble plots of ligand-receptor pairs mediating T cell interactions with other cells. **(D)** Ranking of the importance of each ligand-receptor contribution to the MIF signaling pathway. **(E)** The communication pattern of MIF signaling pathway in different cell clusters. **(F)** Expression levels of receptor-ligand pairs in the MIF signaling pathway.

The relationship between T cells and other immune cells in the RA microenvironment was analyzed using the cellchat package, revealing that T cells communicated with monocytes, NK cells, B cells, plasmablasts, DCs, and pDCs (**Figures 3A, B**). The MIF signaling pathway known to regulate immune responses and inflammation, plays a significant role in the RA microenvironment potentially impacting immune regulation and disease progression. **Figures 3C–E** illustrate that T cells, acting as signal transmitters, mainly interact with other immune cells through the MIF signaling pathway and its associated ligand-receptor pairs (MIF-CD74 + CXCR4 and MIF-CD74 + CD44). Notably, the CD74-CXCR4 pair contributes the most to this pathway, suggesting it may be crucial for promoting the crosstalk between T cells and other immune cells in the RA microenvironment. Furthermore, the MIF-induced ligand CD74 was highly expressed across these immune cell populations (**Figure 3F**) and is likely closely linked to the developmental differentiation of T cells, meriting further investigation. Collectively, these findings underscore that T cells influence other immune cells in the RA microenvironment through a cytokine-mediated paracrine mechanism (16–19).

### 3.3 Trajectory of T cells

**Figure 4:**
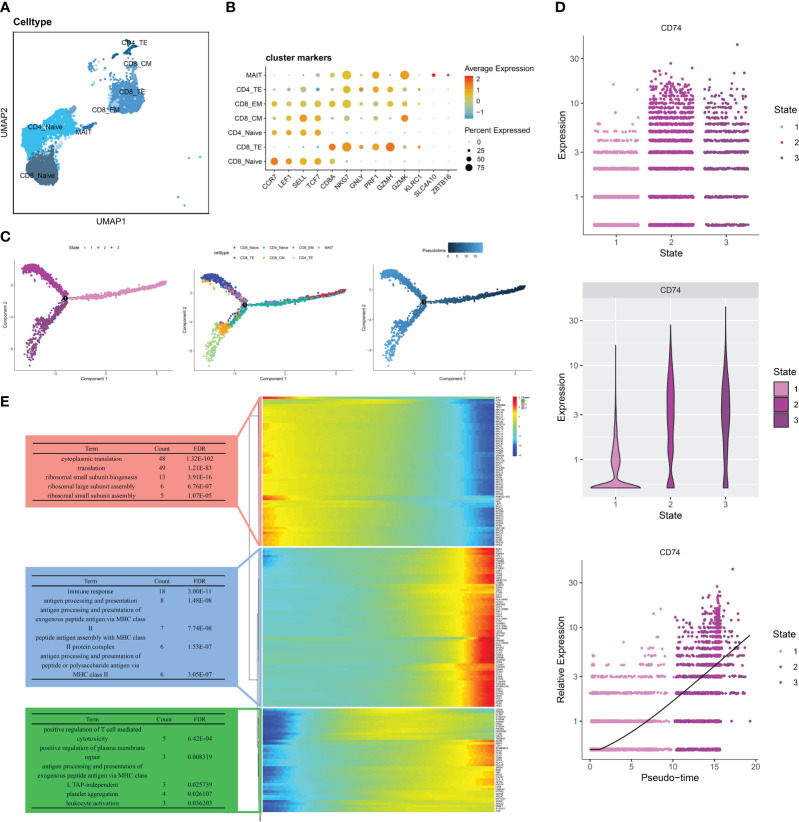
Pseudo-time analysis of T cells in RA PBMCs. **(A)**UMAP of seven T cell subtypes. **(B)** Bubble plots show the expression of marker genes in the 7 T cells subtypes. **(C)** Trajectory plots showing the development of T cells. **(D)** Dynamic expression of ligand CD74 in T cells along pseudo time. **(E)** Heat map showing the expression of dynamic genes in pseudo time and BP enrichment analysis of different states.

Based on the marker genes of T cell subtypes (20), we divided T cells into the following clusters: CD8_Naive (LEF1, CCR7, SELL, TGF7), CD8_TE (GZMH, PRF1, GNLY, NKG7,CD8A), CD4_Naive (LEF1, CCR7, SELL, TGF7), CD8_CM (GZMK, CCR7, TCF7,SEEL), CD8_EM (GZMK, NKG7), CD4_TE (GZMH, PRF1, GNLY, NKG7), and MAIT (SLC4A10, ZBTB16) (**Figure 4A**). Different T cell subtypes express different marker genes (**Figure 4B**). We divided the seven T cell subtypes into three differentiated states by pseudo-time trajectory analysis. The number of T cell subtypes varied in different states. CD8_Naive and CD4_Naive cells were mainly enriched in the early developmental stage of T cells and mainly differentiated into the first cell state; CD8_EM, MAIT, and CD8_TE cells were in the second cell state; and CD8_CM and CD4_TE cells were in the third cell state (**Figure 4C**). Gene changes as T cells developed were divided into three groups associated with cytoplasmic translation, immune response, and positive regulation of T cell-mediated cytotoxicity, respectively (**Figure 4E**). **Figure 4D** shows that the distribution of the MIF-induced ligand CD74 gradually increased with changes over the differentiation period, which indicates that in the RA immune microenvironment, the role of T cells on other immune cells gradually increases as T cells develop.

### 3.4 Identification of risk genes associated with RA

**Figure 5:**
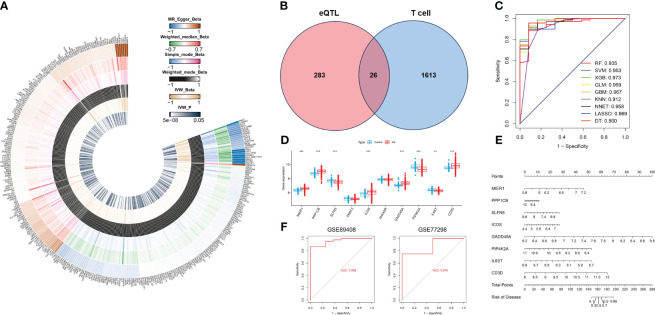
Identification of T cell-related diagnostic features and construction and verification of nomogram models. **(A)** Heatmap showing Beta and IVW-P values of five MR methods for 309 genes. **(B)** Venn diagram of T cell-related genes and RA causal genes. **(C)** ROC curves for nine machine learning models. **(D)** Box plot of differential expression of T cell-related genes in healthy individuals and patients with RA.**(E)** A nomogram model describing T cell-related diagnostic features.**(F)** Validation of the diagnostic model based on eight T cell-related diagnostic features.^*^ *P* < 0.05, ^**^ *P* < 0.01, ^***^ *P* < 0.001.

We downloaded eQTL data for 19,942 genes from blood samples to identify RA-related eQTLs. After harmonizing the SNPs from each eQTLs with the RA GWAS datasets, we identified 24,736 genome-wide SNPs. These SNPs had an F statistic that exceeded 10, indicating a low risk of weak-instrument bias. Following clumping, 5428 genes remained for the MR analysis. Using the IVW method, we identified 309 genetically significant genes associated with RA. Specifically, the increased expression of 153 genes was significantly associated with an increased risk of RA, whereas the increased expression of 156 genes was significantly associated with a decreased risk of RA. (**Figure 5A**, **Supplementary File 2**).

### 3.5 Screening of T cell-related diagnostic features and construction of nomogram model

Based on the single-cell transcriptome analysis of RA, 1639 T cell marker genes were selected for intersection with 309 genes causally associated with RA (**Figure 5B**). We obtained a total of 26 genes, which were used to construct nine machine-learning models. The XGB machine learning model displayed the highest AUC (**Figure 5C**) and exhibited relatively low residuals (**Supplementary Figures S1A, B**). Based on root mean square error (RMSE), we obtained the top 10 significant genes for each machine learning model, and ranked them by importance (**Supplementary Figure S1D**). We selected the top ten genes (MIER1, PPP1CB, SLFN5, PRRT3, ICOS, ANXA2R, GADD45A, PIP4K2A, IL6ST, and CD3D) from the XGB for further analysis. The expression of these 10 genes was analyzed in whole blood samples from healthy individuals and RA patients using the Wilcoxon test in the GSE93272. Compared to healthy individuals, the expression of MIER1, PPP1CB, ICOS, GADD45A, and CD3D was up-regulated in RA, while the expression of SLFN5, PIP4K2A, and IL6ST was down-regulated (**Figure 5D**, **Supplementary Figure S1C**). These genes were defined as T cell-related diagnostic features.

To estimate the risk of RA in 232 patients, we constructed a nomogram model by eight T cell-related diagnostic features (MIER1, PPP1CB, ICOS, GADD45A, CD3D, SLFN5, PIP4K2A, and IL6ST) (**Figure 5E**). The calibration curve showed that the nomogram model accurately predicted RA (**Supplementary Figure S1E**). DCA results showed that the nomogram model had clinical application significance (**Supplementary Figure S1F**). Meanwhile, the predictive power of the model was confirmed by the clinical impact curves (**Supplementary Figure S1G**). We validated nomogram model with external data and obtained AUCs of 0.958 and 0.875 for GSE89408 and GSE77298, respectively (**Figure 5F**), which further demonstrated that our diagnostic model was effective in distinguishing between RA and healthy individuals.

### 3.6 Identification of T cell patterning and analysis of immune cell infiltration

**Figure 6:**
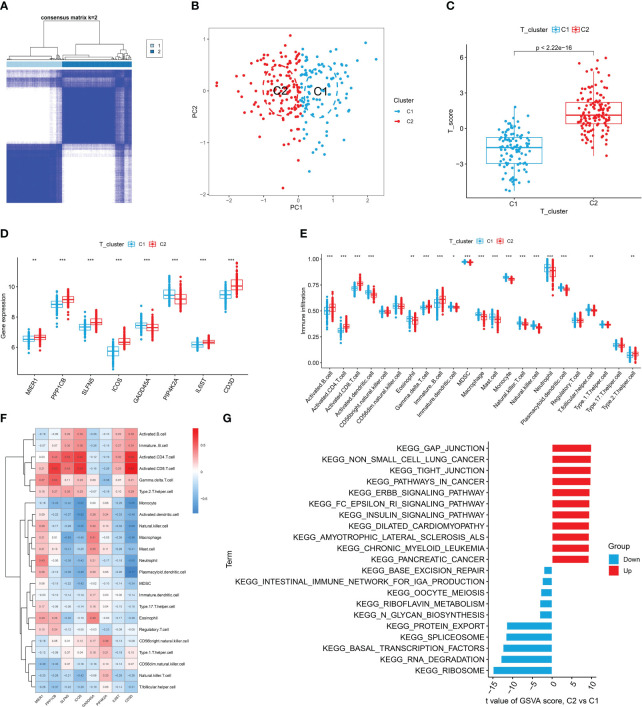
Identification, immune cell infiltration analysis, and GSVA of T cell patterns. **(A)** Consensus clustering matrix of eight T cell-related diagnostic features. **(B)** PCA showed a valid distinction between the two T cell patterns. **(C)** Differences in T cell scores between Cluster 1 and Cluster 2. **(D)** Differential expression of eight T cell-related diagnostic features according to T cell pattern. **(E)** The box plot shows two T cell patterns of immune cell infiltration. **(F)** Correlation heat map of immune cells with eight T cell-related diagnostic features. **(G)** Differential pathways in 2 T cell clusters ^*^ *P* < 0.05, ^**^ *P* < 0.01, ^***^ *P* < 0.001.

To investigate the different patterns associated with T cells in RA, we conducted a consensus cluster analysis of patients with RA based on eight T cell-related diagnostic features. We divided RA into two distinct T cell clusters (Cluster 1 and Cluster 2) (**Figure 6A**, **Supplementary Figure S2A**). PCA results showed a clear distinction between the two T cell patterns (**Figure 6B**). As the result shown, Cluster 2 had higher T cell scores than Cluster 1 (**Figure 6C**). Additionally, we used external datasets GSE89408 and GSE77298 for validation, confirming that the clustering and T cell pattern score results coincided with those training set (**Supplementary Figures S2B, C**). Subsequently, we analyzed the differential expression of eight T cell-related diagnostic features in the two T cell patterns. Compared with Cluster 1, the expression of MIER1, PPP1CB, SLFN5, ICOS, IL6ST, and CD3D was up-regulated in Cluster 2, whereas that of GADD45A and PIP4K2A were downregulated (**Figure 6D**, **Supplementary Figure S2D**). The results of immune cell infiltration showed that activated DCs, eosinophils, MDSC, neutrophils, mast cells, immature dendritic cells, macrophages, monocytes, natural killer T cells, natural killer cells, pDCs, and T follicular helper cells were increased in Cluster 1. In Cluster 2, gamma delta T cells, activated CD4+ T cells, activated CD8+ T cells, activated B cells, immature B cells, and type 2 T helper cells were relatively greater in Cluster 1 (**Figure 6E**). **Supplementary Figure S2E** revealed that Cluster 2 showed high expression in both activated CD4+ T cells and CD8+ T cells, indicating the significant role of T cells in RA as indicated in previous analyses. The correlation between 8 T cell-related diagnostic features and immune cells was determined, suggesting that T cell-induced immune dysfunction in RA may be mainly related to CD3D, ICOS, and PPP1CB (**Figure 6F**). The GSVA results showed that Cluster 2 was mainly related to cancers such as non-small cell lung cancer, pathways in cancer, and pancreatic cancer. While Cluster 1 was enriched in signaling pathways such as those for ribosome, RNA degradation, and basal transcription factor (**Figure 6G**).

### 3.7 Clinical correlation analysis

**Figure 7:**
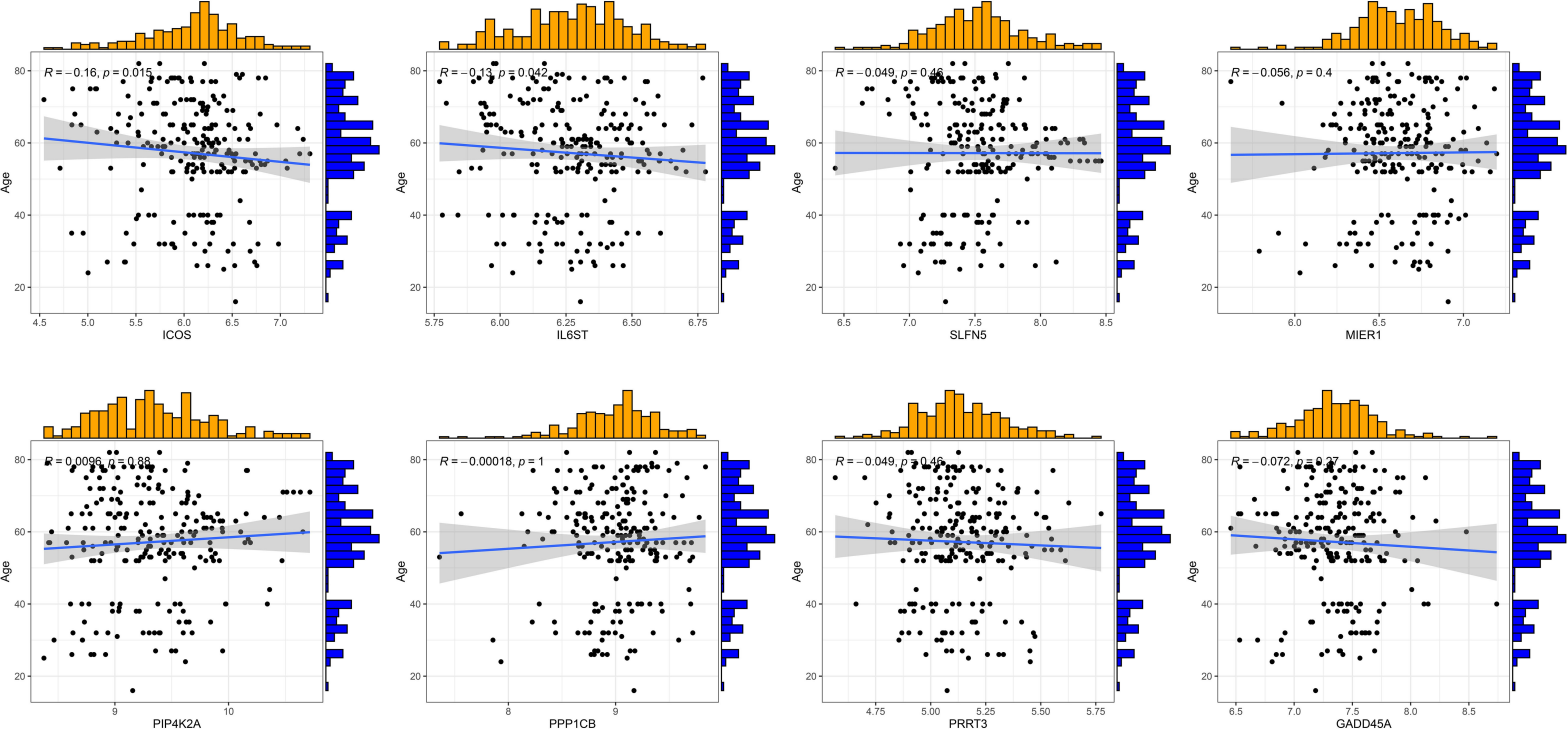
Correlation analysis of eight T cell-related diagnostic features with age of patients with RA.

RA can develop at any age, with the peak incidence occurring between 30 and 60 years. The disease predominantly affects females more than males. We conducted clinical correlation analyses to determine if there were age and gender differences among RA patients with different T cell patterns and to assess if there were any significant correlations between T cell-related diagnostic features and the patients’ ages. The results indicated no significant differences in age distribution between the two different T-cell patterns, nor in the gender proportions (**Supplementary Figures S3A-C**). ICOS and IL6ST were found to be negatively correlated with the age of RA patients, while other T cell-related diagnostic features showed no significant age correlations (**Figure 7**). Therefore, we speculate that age and gender may not be considered when distinguishing RA patients by T cell patterns, though the variations in ICOS and IL6ST expression warrant further investigation.

## 4 Discussion

T cells are one of the important participants in the regulatory network of the immune microenvironment of RA. A variety of cytokines released by different T cell subsets are involved in the inflammatory microenvironment of RA (21).For example, Th17 cells can promote macrophages, synoviocytes and other cells to secrete a large number of inflammatory factors such as IL-1β and IL-6 by producing IL-17 and also contribute to the production of CXCL1, CXCL2, CXCL8 and other chemokines (4, 22). Overactivation of T cells is a factor that triggers maintains RA (1). Therefore, understanding the molecular mechanisms associated with T cells from multiple aspects will help to discover more immunotherapy methods for RA. In recent years, GWAS of RA have shown multiple genomic regions significantly associated with the disease (23, 24). However, the molecular mechanisms underlying risk associated with most sites remain to be explored. At the same time, the basic characteristics of immune cells in RA patients have been fully studied by means of scRNA-seq analysis, and the classification of T cell subsets in RA patients and the regulatory network of interactions between immune cells and T cells have gradually become clear (25). In this study, we systematically elucidated the influence of T cells on the RA microenvironment by constructing a T cell-associated diagnostic model.

This study analyzed the cellular heterogeneity of PBMCs in RA patients and identified seven immune cell types. The results of our cell-cell communication analyses showed that T cells communicated directly with immune cells in the RA microenvironment via paracrine signaling. T cells communicate with other cells primarily through the MIF signaling pathway. Ligand receptors for CD74-CXCR4 play a major role in cell-cell communication networks. MIF, a multi-acting inflammatory cytokine, is closely related to the pathological mechanism of RA and can induce the secretion of inflammatory cytokines and molecules in degraded tissues (26, 27). MIF can affect the microenvironment in autoimmune diseases through autocrine and paracrine pathways through receptors such as CD74 and CXCR4 (28, 29). Studies have shown that the shedding of CD74 extracellular domain may lead to a marked increase in serum CD74 levels in RA patients (29).Therefore, we hypothesized that the activation of MIF signaling pathway (MIF-CD74 + CXCR4) in the microenvironment of RA patients affects the communication between T cells and other immune cells, and that the high expression of CD74 in the cell population warrants further investigation as a key mediator of cell signaling. This intercellular communication has the potential to influence the immune landscape of RA. By interacting with other immune cells, T cells become more fully involved in immune system activation and disease progression.

We divided the T cells into seven subtypes: CD8_Naive, CD8_TE, CD8_CM, CD8_EM, CD4_Naive, CD4_TE, and MAIT. In the analysis of pseudo-time trajectories of T cells, dynamic expression of the MIF-induced ligand CD74 was positively correlated with differentiation state, suggesting that as T cells differentiate, their influence on immune cells in the RA microenvironment is progressively increased through a paracrine mechanism. Given the critical role of T cells in inducing the RA immune response, they are recruited to the synovial tissue of the joint via the blood stream and interact with antigen-presenting cells. By analyzing the communication between T cells and other immune cells in peripheral blood, we explored T cells’ potential role in disrupting the immune microenvironment through the MIF pathway and paracrine mechanisms. Subsequently, pseudo-time trajectories analysis showed the differentiation process of T cells and the functional enrichment changes at different developmental stages, further highlighting the importance of T cells in the pathogenesis of RA. Understanding these complex interactions provides new insights for developing targeted immunotherapeutic strategies. Additionally, this analysis also provides robust data support for constructing RA-related diagnostic and prognostic models based on T cells in this study.

To further improve the understanding of the effect of T cells on the diagnosis and prognosis of RA, we obtained eight T cell-related diagnostic features by combining machine learning and MR (ICOS, IL6ST and PPP1CB were risk factors for RA, while GADD45A, CD3D, SLFN5, PIP4K2A and MIER1were protective factors for RA), which were constructed a T cell-related diagnostic model. The external datasets verified the efficiency of the diagnostic model, which was effective in distinguishing RA patients between healthy individual. Inducible T cell co-stimulator (ICOS) is mainly present on the surface of activated T cells and can enhance the differentiation and function of inflammatory T cells (30, 31). Studies have found high levels of ICOS+ T cells and ICOS ligand (ICOSL) in synovial fluid of RA patients joints (32). Vincent et al. (33) found that ICOS is a key co-stimulatory pathway controlling the induction and maintenance of colliology-induced arthritis (CIA), and there is a potential overlapping relationship between ICOS signal transduction, T cell glycolysis, and joint inflammation. We speculate that ICOS at these overlapping points could serve as a therapeutic target for RA. Growth arrest and DNA damage protein 45A (GADD45A) could promote the activation of naïve T lymphocytes into Th1 cells in adaptive immune responses by inducing the activation of p38 MAPK in DCs (34). Li et al. (35) studied polymorphisms in the GADD45A promoter region in RA patients and found that the severity of RA could be explained in part by the polymorphic expression of GADD45A.CD3D is involved in the formation of T cell receptor/CD3 complex, as well as T cell development and signal transduction. And the loss of CD3D is associated with T-cell development and signal transduction (36). Additionally, CD3D has been confirmed to be involved in abnormal activation of immune-related pathways in T lymphocytes in epigenetic and genomic analyses (37). These studies suggest that CD3D may be associated with overactivation of T cells in the RA microenvironment. Schlafen family member 5 (SLFN5) was found to have high basal expression levels in resting T cells and was downregulated after T cell activation, suggesting that SLFN5 may be involved in the maintenance of T cell quiescence (38). At the same time, our bulk data showed low expression of SLFN5 in patients with RA, suggesting that SLFN5 may be involved in RA progression by regulating T cell activation. Alessandro et al. (39) proposed that phosphatidylinositol-5-phosphate (PtdIns5P)-4-kinases (PIP4Ks) specifically control the growth and activity of regulatory T cells (Tregs) isolated from human blood. Inhibition of PIP4K (PIP4K2B and PIP4K2C) could reduce signaling of inflammatory pathways such as PI3K and MAPK in Tregs, thereby reprogramming the identity of human Tregs while increasing conventional T-cell signaling and helper T-cell differentiation, potentially enhancing overall immune activity. Our results indicate that PIP4K2A is expressed at low levels in the PBMCs of patients with RA. Whether PIP4K2A also affects the microenvironment of RA by participating in the immune function of Tregs needs to be further explored. The interleukin 6 cytokine family signal transducer (IL6ST) is a signal transduction of activating inflammation-related signaling pathways such as JAK/STAT and MAPK/PI3K/ERK, and plays a key role in immune function (40). At present, IL6ST has been confirmed to be associated with the risk of autoantibody positive RA, and is a risk allele for RA (41). Mesoderm induction early response 1 (MIER1) is a transcription regulator activated by fibroblast growth factors (42). Mier1-α, a novel estrogen receptor-binding protein, has been studied mainly in invasive breast cancer (43) and has also been identified as a key regulator of liver regeneration (44). PP1-beta-catalytic subunit (PPP1CB), as one of the three catalytic subunits of protein phosphatase 1 (PP1), has been reported to be responsible for transient Ca2+ elevation and enhanced cell shortening in cardiomyocytes. It can also regulate adipocyte differentiation by targeting the transcription factor C/EBPδ (45, 46). However, MIER1 and PPP1CB, as the T cell-related diagnostic genes screened in this study, have not been reported to be related to RA so far, which needs to be further explored. In summary, MIER1, PPP1CB, ICOS, GADD45A, CD3D, SLFN5, PIP4K2A, and IL6ST are noteworthy T cell diagnostic model indicators of RA.

We divided the patients with RA into two groups of T cell patterns, Cluster 1 and Cluster 2, based on eight T cell-related diagnostic features. Immune infiltration analysis showed that the proportion of activated CD4+/CD8+T cells and T cell score in Cluster2 were higher than those in Cluster1. T cells are one of the key target cells for tumor immunotherapy. The composition and status of T cells can be affected by the microenvironment of different types of tumors (47). GSVA results showed that Cluster 2 was mainly related to cancers such as non-small cell lung cancer, pathways in cancer, and pancreatic cancer, indicating that the pathogenesis of Cluster 2 patients may be related to abnormal signaling of T cells in the tumor microenvironment. Clinical correlation analysis revealed no significant correlation between age and sex distribution in the two T cell models. However, ICOS and IL6ST are negatively correlated with the age of RA patients, suggesting that these two genes may be involved in the regulation of the pathological process of age-related RA.

Zhang et al. (48) constructed a single-cell atlas of RA synovial tissue comprising over 314,000 cells, providing new insights into RA pathology and heterogeneity. In contrast, we performed cell-cell communication and pseudo-time trajectories analyses on T cells in peripheral blood monocytes of RA patients for the first time, offering a unique perspective on the molecular characteristics of immune cells in RA via peripheral blood, a critical component of the RA immune system. Additionally, by unprecedentedly integrating multiple datasets, we identified convincing RA diagnostic genes and further constructed a diagnostic model of RA T cells building on previous exploration of RA diagnostic models. Although the T cell-related diagnostic model developed in this study is helpful for identifying RA patients and predicting prognosis, it still has some limitations. Firstly, the diagnostic model was built based on the publicly available dataset, and the sample types of RA patients were not rich enough, so more samples of different types are needed to verify the expression levels of T cell diagnostic genes. Secondly, a large number of clinical trials are needed to verify the accuracy of the prediction model. In addition, there is a lack of molecular biology experiments to verify the mechanism of T cell diagnostic genes in RA. In addition, the GWAS and eQTL data in this study were obtained from populations of European origin, and the applicability to populations of other ethnicities needs to be further investigated. And the results were not validated at the protein level.

In summary, this study integrated eQTL, GWAS, scRNA-seq, and bulk RNA-seq multiple datasets to explore the T cell profile and microenvironment in RA and identify T cell-related diagnostic features. In this study, the abnormal characteristics of T cells in RA patients were analyzed in terms of intercellular communication, cell developmental trajectories, and functional enrichment. In addition, our T cell-related diagnostic model showed good diagnostic performance. Our findings deepen our understanding of the regulatory network of immune cells in the RA immune microenvironment and explore the potential role of T cells in the disease progression. We hope that this comprehensive evaluation of models will help advance the development of precision immunotherapies for RA.

## Data availability statement

The datasets presented in this study can be found in online repository. The names of the repository and accession numbers can be found in the article/**Supplementary Material**.

## Author contributions

QD: Writing – original draft. QX: Writing – original draft. YH: Writing – original draft. HZ: Writing – original draft. XH: Writing – original draft. CN: Writing – original draft. ZT: Writing – original draft. HL: Writing – original draft. PZ: Funding acquisition, Writing – review & editing. JL: Data curation, Formal analysis, Funding acquisition, Visualization, Writing – review & editing.

## Supplementary material

Supplementary Figure 1 Construction and verification of nomogram model. **(A)** Reverse cumulative distribution of residuals of nine machine learning models. **(B)** Residual boxplot of nine machine learning models for diagnostic gene selection. **(C)** T cell-related diagnostic features and expression heat maps in healthy individuals and patients with RA. **(D)** Features important for nine machine learning models. **(E)** The calibration curve of the nomogram model. **(F)** DCA curves. **(G)** Evaluation of the clinical impact of nomogram modelling using clinical impact curves. ^*^ *P* < 0.05, ^**^ *P* < 0.01, ^***^ *P* < 0.001.

Supplementary Figure 2 Classification of T cells and analysis of immune cell infiltration. **(A)** Representative cumulative distribution function curve. **(B)** Consensus clustering matrix for the external validation dataset. **(C)** Differences in T cell scores between Cluster 1 and Cluster 2. **(D)** Differential expression heatmaps of eight T-cell related diagnostic features according to T-cell patterns. **(E)** Boxplots of immune infiltration analysis for the two T cell patterns. ^*^ *P* < 0.05, ^**^ *P* < 0.01, ^***^ *P* < 0.001.

Supplementary Figure 3 Correlation analysis of two T cell patterns and eight T cell-related diagnostic features with age and sex. **(A)** Differences in age distribution between the two T cell clusters. **(B, C)** Differences in sex distribution between the two T cell clusters.
