# Supplementary figures and images for: Integrated analysis of single-cell RNA-seq, bulk RNA-seq, Mendelian randomization, and eQTL reveals T cell-related nomogram model and subtype classification in rheumatoid arthritis
Source: Front Immunol. 2024 Jun 19;15:1399856. doi: 10.3389/fimmu.2024.1399856 (PMC11219584; doi:10.3389/fimmu.2024.1399856)

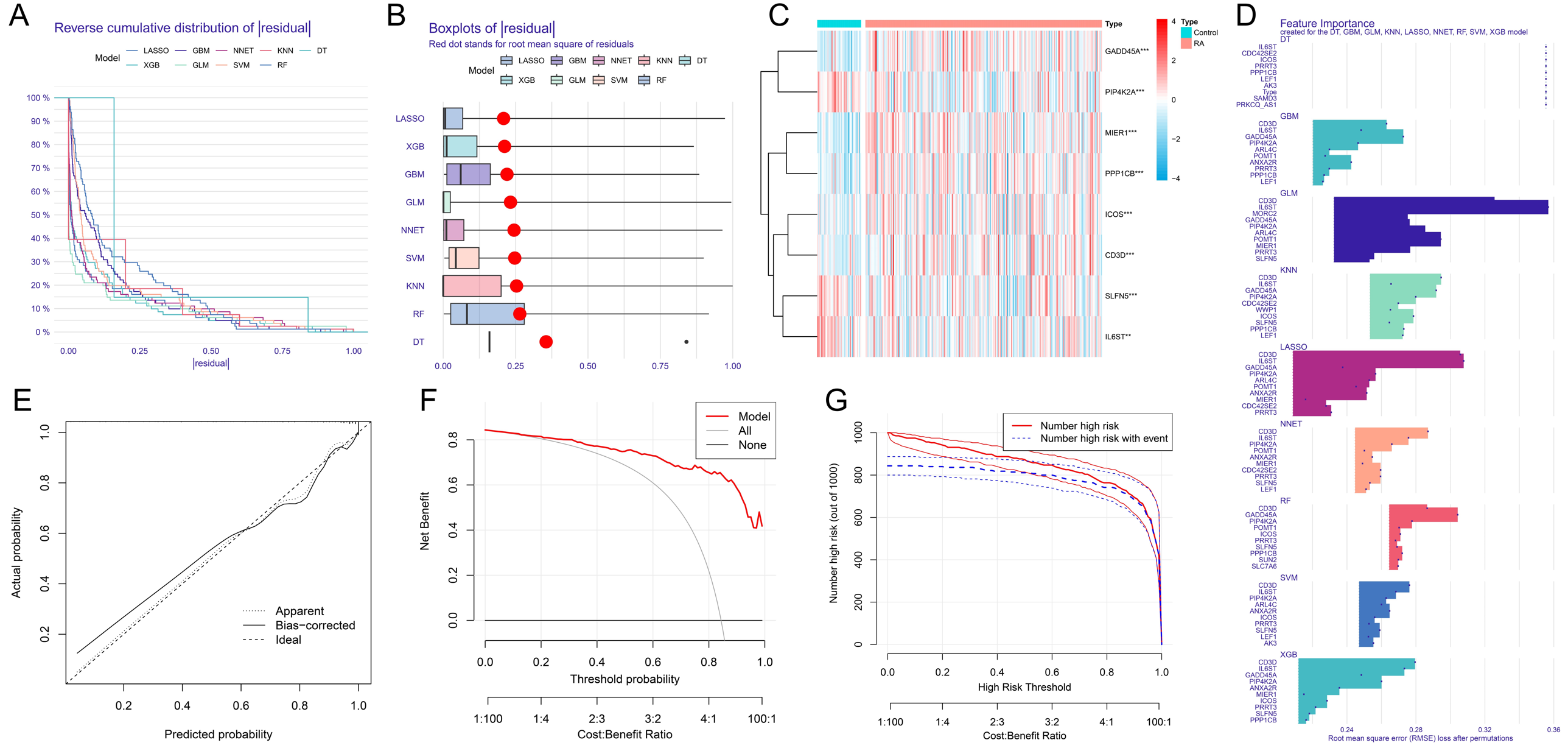

Supplement: Supplementary Figure 1 — Construction and verification of nomogram model. (A) Reverse cumulative distribution of residuals of nine machine learning models. (B) Residual boxplot of nine machine learning models for diagnostic gene selection. (C) T cell-related diagnostic features and expression heat maps in healthy individuals and patients with RA. (D) Features important for nine machine learning models. (E) The calibration curve of the nomogram model. (F) DCA curves. (G) Evaluation of the clinical impact of nomogram modelling using clinical impact curves. * P < 0.05, ** P < 0.01, *** P < 0.001. [file Image_1.jpeg]

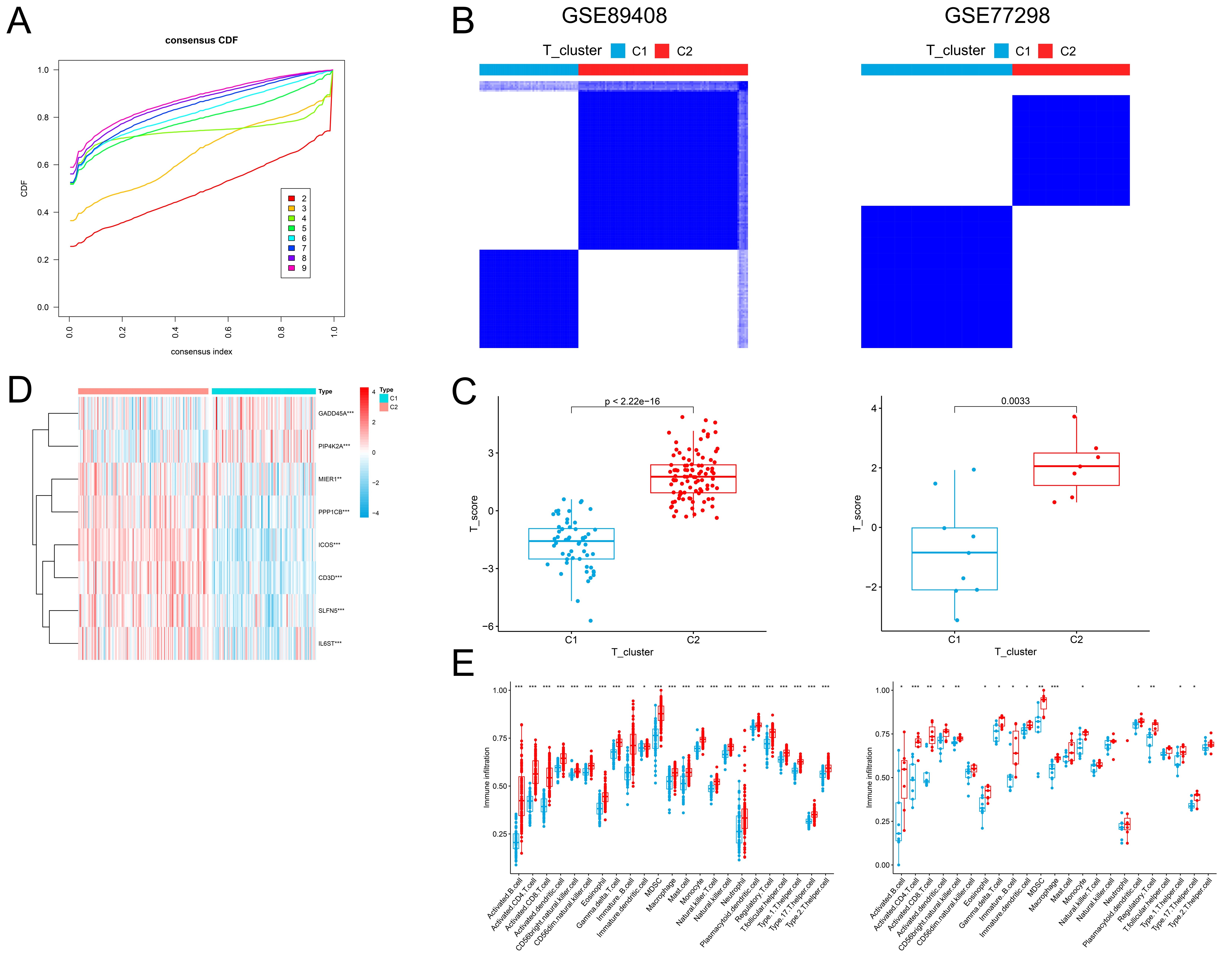

Supplement: Supplementary Figure 2 — Classification of T cells and analysis of immune cell infiltration. (A) Representative cumulative distribution function curve. (B) Consensus clustering matrix for the external validation dataset. (C) Differences in T cell scores between Cluster 1 and Cluster 2. (D) Differential expression heatmaps of eight T-cell related diagnostic features according to T-cell patterns. (E) Boxplots of immune infiltration analysis for the two T cell patterns. * P < 0.05, ** P < 0.01, *** P < 0.001. [file Image_2.jpeg]

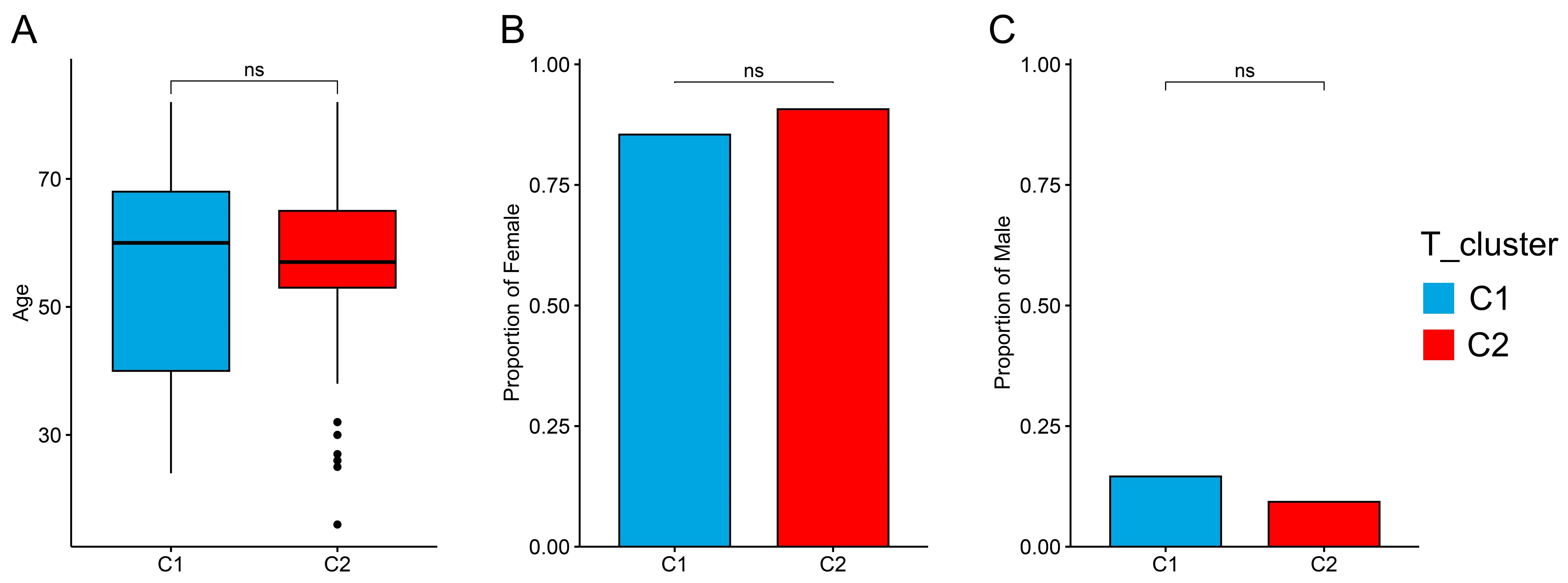

Supplement: Supplementary Figure 3 — Correlation analysis of two T cell patterns and eight T cell-related diagnostic features with age and sex. (A) Differences in age distribution between the two T cell clusters. (B, C) Differences in sex distribution between the two T cell clusters. [file Image_3.jpeg]
